# Candidate genes associated with reproductive traits in rabbits

**DOI:** 10.1007/s11250-024-03938-8

**Published:** 2024-03-05

**Authors:** Mostafa Helal, Jana Sameh, Sama Gharib, Rana M. Merghany, Milena Bozhilova-Sakova, Mohamed Ragab

**Affiliations:** 1 Department of Animal Production, Faculty of Agriculture, Cairo University, Giza, 12613 Egypt; 2 Biotechnology Program, Faculty of Agriculture, Cairo University, Giza, 12613 Egypt; 3 Department of Pharmacognosy, National Research Centre, Giza, 12622 Egypt; 4 Agricultural Academy, Institute of Animal Science, Kostinbrod, 2232 Bulgaria; 5 Poultry Production Department, Faculty of Agriculture, Kafrelsheikh University, Kafrelsheikh, Egypt; 6 Animal Breeding and Genetics Department, National Institute for Agricultural and Food Research and Technology (INIA), Madrid, 28040 Spain

**Keywords:** Rabbit breeding, Prolificacy, MAS, Candidate genes, Polymorphism

## Abstract

In the era of scientific advances and genetic progress, opportunities in the livestock sector are constantly growing. The application of molecular-based methods and approaches in farm animal breeding would accelerate and improve the expected results. The current work aims to comprehensively review the most important causative mutations in candidate genes that affect prolificacy traits in rabbits. Rabbits are a source of excellent-tasting meat that is high in protein and low in fat. Their early maturity and intensive growth are highly valued all over the world. However, improving reproductive traits and prolificacy in rabbits could be very tricky with traditional selection. Therefore, traditional breeding programs need new methods based on contemporary discoveries in molecular biology and genetics because of the complexity of the selection process. The study and implementation of genetic markers related to production in rabbits will help to create populations with specific productive traits that will produce the desired results in an extremely short time. Many studies worldwide showed an association between different genes and productive traits in rabbits. The study of these polymorphisms and their effects could be useful for molecular-oriented breeding, particularly marker-assisted selection programs in rabbit breeding.

## Background

Rabbits are animals that possess valuable characteristics such as high fertility, early maturity, rapid growth, and high feed efficiency (Bindu et al., 2012; Helal, 2019). They are an important resource in the meat industry, as their meat has good quality and low fat content (El-Sabry et al., 2021; Wang et al., 2022).

There are many non-genetic factors influencing reproductive traits in rabbits. Environmental conditions such as temperature and photoperiod can directly affect reproductive processes (El-Sabry et al., 2021). Nutrition is another crucial environmental factor that impacts reproductive traits. Inadequate nutrition can lead to delayed sexual maturation, reduced fertility, and decreased reproductive success. Social interactions and social structure within a species can also influence reproductive traits (Rödel, 2022).

Genetic improvement of reproductive traits and prolificacy, particularly in local rabbit breeds, is a crucial step in improving their performances in general (Gootwine, 2020). Conventional selection is a slow and uncertain method for improving reproductive traits because they are sex-limited and most of them have low heritability levels, so the genetic gains are expected to be low (Sosa-Madrid et al., 2020). For instance, the response to selection for litter size in rabbits is estimated at 0.1 kits per generation (Khalil and Al-Saef, 2008). Therefore, traditional breeding programs need new methods based on contemporary discoveries in molecular biology and genetics because of the complexity of the selection process and the size of populations (Getmantseva et al., 2020). This can be achieved by including genomic information in breeding programs to accelerate the rate of genetic changes using marker-assisted selection (MAS) programs (Bozhilova-Sakova et al., 2022; Helal and El-Gendy, 2023).

The advancement of genomics has revolutionized the ability to conduct complete sequencing of numerous livestock genomes, enabling the exploration of genetic architecture underlying complex traits. Recent investigations have generated a growing body of evidence clarifying the pivotal role of polymorphisms in dictating variations in quantitative traits of substantial impact (Milan et al., 2000; Khalil, 2020; Helal et al., 2021). It has become possible to map a sizable number of quantitative trait loci (QTL) linked to changes in observable phenotypic features within livestock genomes. These methodologies have facilitated the elucidation of gene sets that play fundamental roles in crucial biological functions, thereby driving efforts to bridge the considerable gap existing between genotype and phenotype (Carneiro et al., 2014; Zhou et al., 2018).

In rabbits, laparoscopy allows for the simultaneous measurement of ovulation rate, number of implanted embryos, and litter size within a single gestation period without adversely affecting the resulting litter size (Santacreu et al., 1990). However, in rabbit breeding the most commonly used criterion for selecting maternal lines is the litter size, either at birth or at weaning (Garreau et al., 2004). This particular characteristic holds even greater significance compared to other highly reproductive species (Armero and Blasco, 1993). There are many genes linked to different reproductive traits in animals (An et al., 2009). In this context, genes affecting uterine capacity and other different reproductive traits were investigated in two divergently selected rabbit lines. The lines were selected for uterine capacity (Argente et al., 2003). The results showed that many genes had a large effect on the number of implanted embryos and embryo survival, and other genes of relatively moderate effect on ovulation rate, fetal survival, and prenatal survival. Polymorphisms in these genes may cause changes in the different traits. The study of these polymorphisms in different genes is useful for molecular-oriented breeding, particularly marker-assisted selection programs (An et al., 2010). When practicing selection, it is crucial to analyze the causative genes through markers associated with those genes (Helal, 2019). This analysis can contribute valuable benefits for improving quantitative traits such as litter size, milk yield, growth, and meat production (An et al., 2013; Cardoso et al., 2014; Helal et al., 2021).

The candidate gene approach can be used to identify the effect of a single nucleotide polymorphism (SNP). Moreover, candidate gene investigations do not require the inclusion of extensive pedigrees consisting of both affected and unaffected individuals. Instead, they can be conducted using unrelated individuals or with a limited number of families. Additionally, candidate gene studies are particularly well-suited for identifying genes that underlie the complex inheritance of economically important traits (Wang et al., 2019; Welk, 2019).

Worldwide, there is extensive work addressing the association between SNPs in candidate genes and reproductive traits in goats, sheep, and swine (Bidanel, 2013; Abdoli et al., 2016; Mishra et al., 2017; Nenova et al., 2023). There are very few major genes that are associated with litter size in rabbits. Therefore, the current work aims to comprehensively review the most important causative mutations in candidate genes that affect prolificacy traits in rabbits.

### Progesterone receptor gene (PGR)

The *PRG* gene (ENSOCUG00000014693) resides on chromosome 1 (115,601,359 − 115,672,617 F), and has 4 transcripts encoding for proteins ranging between 761 and 930 amino acids. The product of this gene interacts with the progesterone hormone to play a vital role in fetus maintenance. It has also an indirect effect on reproduction by altering maternal behavior (Benedek et al., 2020). There are two isoforms of progesterone receptors (PR-A and PR-B). The PR-A isoform is essential to stimulating reproductive responses that are important for fertility, while PR-B is required for stimulating normal proliferative responses of the mammary gland to progesterone (Mulac-Jericevic et al., 2000; Conneely et al., 2002).

In 2006, the *PGR* SNP g.2464 G > A was reported to influence the difference in litter size and its components between high and low rabbit lines selected for uterine capacity (Peiró et al., 2006). Thereafter, the *PRG* gene was genotyped in 598 hybrid rabbits from crossing between low and high divergently selected rabbit lines for uterine capacity for 10 generations (Peiró et al., 2008). After sequencing the gene, five SNPs were detected, one SNP in the PRG premotor region (g.2464 G > A), three SNPs in 5’-UTR of exon 1, and one SNP in exon 7. The allele G of the first SNP was predominant in the high parental line, and the GG genotype had more implanted embryos and more kits than the AA genotype. The GG genotype had 0.36 embryos more than the AA genotype. The same mutation was genotyped in three rabbit breeds (Moshtohor line, V-line, and Gabali) in Egypt (El-Aksher et al., 2017). Although the frequency of the GG genotype was rare (0.04), the allelic frequency of the G allele was 0.38. Nevertheless, the results indicated that the Moshtohor line had high litter traits associated with the GG genotype. Also, PCR-RFLP was used to genotype the same SNP in a local Slovak crossbred rabbit line (Ondruska et al., 2020). The frequency of the G allele was 0.47, and the GG genotype was superior and had higher milk production (*P* < 0.001), litter size (*P* < 0.05), and number of weaned rabbits per litter (*P* < 0.001). However, the average number of stillborn kits per litter was significantly higher (*P* < 0.05) for AA compared to the GG genotype. Total milk yield in 28 days was also significantly higher for the GG genotype in Gabali rabbits, however, had a heterozygous AG genotype with a significantly higher litter size at birth and weaning (Ramadan et al., 2020). In Californian rabbits, the g.2464G > A mutation was genotyped in 60 individuals from high litter size and low litter size groups (Kotsyubenko et al., 2017). The frequency of allele G was 0.35 in the high litter-size rabbits and 0.20 in the low litter-size rabbits, but the genotype GG did not exist in any of the two groups, and the SNP did not occur in the low or high litter-size group.

In goats, the PGR gene polymorphism had a significant effect on the litter size of Jining Grey goats (Wang et al., 2009). Similar results for litter size were reported in Nubian goats (Zhang et al., 2021). In pigs, polymorphism in the PGR gene was associated with the number of healthy births (Ding et al., 2021). The *PRG* gene polymorphism was not only associated with reproductive traits, but it was also associated with body weight in rabbits (El-Aksher et al., 2016).

### Follicle-stimulating hormone subunit beta gene (FSHβ)

The *FSHβ* gene (ENSOCUG00000021932) resides on chromosome 1 (169,431,050–169,434,819 F) in rabbits and has three exons and one transcript encodes for a 29 amino acid protein. *FSHβ* gene in rabbits is vital in the synthesis of FSH, which is critical for regulating reproductive functions, including follicular development, spermatogenesis, and steroidogenesis (Molés et al., 2012). The *FSHβ* gene encodes for the beta subunit of FSH, which is necessary for appropriate hormone assembly and secretion (Pooten and Mingala, 2021). The absence of a functional *FSHβ* gene in rabbits may lead to disruptions in reproductive processes and fertility. Moreover, the *FSHβ* in rabbits plays a role in ovulation (Recchia et al., 2021). Despite the *FSHβ* gene in rabbits is considered a highly conserved gene, it has some variants in its structure (Niu et al., 2019).

The gene was re-sequenced and genotyped in 170 Rex rabbits (Niu et al., 2019), and 37 SNPs were detected. Five of them were genotyped, two in exon 1 (g.218 G > A, and g.284 G > T), and three in exon 3 (g.2779 G > C, g.2908 G > A, and g.2963 G > A). The GG genotype of the g.284 G > T mutation and the AA genotype of g.2908 G > A mutation were significantly associated (*P* < 0.05) with the total number born, and number born alive. Also, the GG genotype of g.2963 G > A mutation was superior and was significantly associated (*P* < 0.05) with higher litter weight at birth, total number born, and litter size at 21 days of age.

Several SNPs in the *FSHβ* gene were linked with semen traits (Nikbin et al., 2018), and litter size (Zhang et al. 2011a; An et al. 2015; Aljuaydi et al. 2016; Pooten and Mingala 2021) in goats, litter size in sheep (Li et al., 2019), and sperm quality in Holstein Bulls (Ghasemi and Ghorbani, 2014). Also, *FSHβ* SNPs were associated with different traits including litter size (Qu et al., 2008; Zhang et al., 2010; Wu et al., 2023), and backfat thickness (Zhu et al., 2016) in pigs.

### Oviductal glycoprotein 1 gene (OVGP1)

Another important gene is the *OVGP1* gene (ENSOCUG00000015946), which resides on chromosome 13 (52,788,863 − 52,803,127 F), and has only one transcript encoded for a 475 amino acid protein. The product of this gene is oviductal glycoprotein 1 (OVGP1), which is one of the major components of oviductal fluid (Yu et al., 2019). This fluid provides the ideal environment, including temperature, pH, and oxygen tension, and also controls early pregnancy (Killian, 2004; Saeed et al., 2015).

The promoter and intron 1 region of the *OVGP1* gene was sequenced in 121 animals belonging to four Spanish rabbit lines, including A, H, V, and R lines (Merchán et al., 2007). The sequencing resulted in five SNPs in the promoter, three SNPs, and one INDEL mutation in intron 1. Thereafter, a fragment of 854-bp was re-sequenced (part of the promoter, exon 1, and part of intron 1) in 16 rabbits, 4 rabbits were from the line selected for high uterine capacity and 12 from the line selected for low uterine capacity (Merchán et al., 2009). They also sequenced another fragment (1528-bp) including all the coding sequences as well as the partial 3’UTR in 4 from high litter and 10 rabbits from low lines, and 11 SNPs were identified in exons 3 (2 SNPs), 6 (2 SNPs), 8 (1 SNP), and 11 (6 SNPs). Two mutations in exon 11 were nonsynonymous mutations, the first one was g.12903G > C (Gly454Ala), and the second one was g.12,944 C > G (p.Arg468Gly) and these changes were found to be directly involved in regulating the biological activity of *OVGP1*. There was a strong correlation between g.12,944 C > G SNP and total number of kits born, the number of implanted embryos, and the number of born alive (NBA). Moreover, the SNP 12,944 C > G (exon 11) was genotyped in 331 individual reciprocal cross rabbits generated from crossing between low and high prolific rabbit lines selected for high and low uterine capacity (García et al., 2010). The results revealed that the GG genotype was superior to the CC genotype in early embryos (survival 72 h of gestation). Litter size traits in pigs were reported to be affected by *OVGP1* gene polymorphisms (Niu et al., 2006).

### Tissue inhibitor of metalloproteinase 1 gene (Timp1)

The *Timp1* gene (ENSOCUG00000010718) resides on chromosome X (32,653,959 − 32,658,564 F). It has six exons and two transcripts. The *TIMP1* gene exhibits a conserved structural pattern across various mammalian species, including humans, dogs, mice, and others, with six exons and five introns. Similarly to humans (Clark et al., 1997) and mice (Coulombe et al., 1988), the coding region of the *TIMP1* gene commences at exon 2 and concludes at exon 6, with exon 1 being noncoding. This gene is from the tissue inhibitor of the metalloproteinase (*TIMP*) gene family where the proteins produced from them are natural inhibitors of matrix metalloproteinase (MMPs), which are peptidases and take part in the degradation of the extracellular matrix. This protein can promote cell proliferation in a broad range of cell types and has an antiapoptotic function. Its transcription is greatly inducible in response to cytokines and hormones. This gene also plays roles in embryotropic action and supporting embryonic development (Satoh et al., 1994; Hwang et al., 2000), since TIMPs are considered growth promoters, and apoptosis modulators (Brew et al., 2000).

The *TIMP1* gene has a strong effect on the reproductive traits of rabbits. The TIMP1 gene was sequenced in the parental generation of a second-generation cross between lines with high and low uterine capacity (Estellé et al., 2006). Despite the monomorphism observed in the screened regions, including the coding sequence, proximal promoter, exon 1, intron 1, and exon 2, a quantitative real-time reverse transcription-polymerase chain reaction analysis of *TIMP1* mRNA expression in the oviduct revealed significant differences between high and low lines at 62 h of gestation, coinciding with the presence of rabbit embryos in the oviduct. Argente et al. (2010) sequenced the same gene, including the proximal promoter region, exon 1, intron 1, the 5′ end of exon 2 and the CDS region in high and low rabbit uterine capacity rabbit lines A SNP (g.1423 A > G) was found to be with different frequencies in both lines (0.60 for allele A, and 0.82 for allele G for the high and low lines, respectively). The association was calculated in 598 individuals of F2 hybrid rabbits generated by crossing the high and low lines. The results revealed that the AA genotype has 0.88 additional embryos compared to the GG genotype after 72 h of gestation. This difference reached 2.23 embryos at the implantation stage. However, no significant difference was observed between the genotypes in terms of litter size at birth.

The *TIMP1* gene affects reproductive traits in goats by regulating estrogen secretion and the expression of lambing-related genes in granulosa cells (Hong et al., 2022). Also, it regulates the reproductive cycle in mice (Nothnick, 2000) and embryo implantation in sheep (Pokharel et al., 2020). In humans, the *TIMP1* gene was associated with recurrent spontaneous abortions in Han Chinese couples (Song et al., 2014).

### Prolactin receptor gene (PRLP)

The *PRLR* gene (ENSOCUG00000005274) is the receptor for prolactin. This gene resides on chromosome 11 (56,363,806 − 56,565,013R), and it has 17 exons encoding 7 transcripts. The *PRLP* gene belongs to the same family as the growth hormone receptor gene. Upon binding of prolactin to its receptor (PRLR) the Janus kinase/signal transducer and activator of transcription (JAK/STAT) signaling pathway is activated. The Stat proteins, which are the key participants in this pathway, play a crucial role in modulating cellular responses to various cytokines and growth factors (Binart et al., 2010). The *PRLP* gene is associated with both reproductive and growth traits, as well as affecting milk production and milk characteristics. The PRLP gene controls the prolactin signaling cascade, which in turn regulates water and electrolyte balance, growth, modulates brain function and behavior, and regulates reproductive processes. (Bole-Feysot et al., 1998; Viitala et al., 2006). Prolactin also plays a critical role in the initiation and maintenance of lactation and synthesis of key milk components (Brym et al., 2005). Furthermore, it is worth noting that the *PRLR* gene overlaps with one of the most prominent and robust sweep signals observed within the rabbit genome (Carneiro et al., 2015).

Does’ milk production and reproductive processes are significantly influenced by the maternal genotype. In this regard, the PRLR gene promoter was sequenced in the European wild rabbit, which is a cross of Hungarian wild rabbit and Slovakian wild-caught wild rabbits (Benedek et al., 2023). The length of the sequenced fragment was 1210 bp, in which 4 point mutations (g.407G > A, g.496G > C, g.926T > C, and g.973 A > C), and one microsatellite located at position 574 were detected. The 4 SNPs were segregated into 4 genotypes (AACCCCCC, GGGGTTAA, AAGGTTAC, and GGGGTCAC). The results revealed that the homozygous genotype was associated with high milk production. Furthermore, the short microsatellite repeat (167 bp) was associated with significantly higher milk production. Moreover, the *PRLP* gene was found to be associated with the reproductive seasonality trait between wild and domestic rabbits (Carneiro et al., 2015).

In Small-Tail Han sheep, polymorphisms in the *PRLP* gene were linked with litter size traits (Chu et al. 2007a). Also, there was an associati on between PRLP gene polymorphisms and prolificacy in the Lezhi black goats (Wu et al. 2014a) and in the Jining Grey goat (Zhang et al., 2007). In boar goats, the *RRLP* gene polymorphism was also associated with litter size (Li et al., 2011). In pigs, the coding region of the *PRLR* gene was sequenced and six SNPs were detected, and the results revealed the effect of *PRLP* gene polymorphisms on the ovulation rate (Tomas et al., 2006).

### κ-casein gene (CSN3)

The *CSN3* gene (ENSOCUG00000015167) resides on chromosome 15 in rabbits (79,462,339 − 79,655,733R) and has five exons and two transcripts. The 13 kb κ-casein gene has five exons, and codes for a protein called kappa-casein, which is essential for micelle formation and stabilization (Hiripi et al., 2000). This gene was identified in rabbits in 1993 (Bösze et al., 1993). In 1998, it was revealed by Hiripi et al. (1998) that it has two alleles (A and B) that were translate to identical κ-casein. The sequence of the gene in rabbits is closer to the ovine than to the mouse sequence (Bösze et al., 1993).

In rabbits, a genotype at the κ-casein locus that was either AA or AB and had their reproductive features recorded at birth and at weaning for a total of 743 litters. These were the offspring of 18 males with the genotype AA and 77 females with the k-casein heterozygous genotype AB. A significant correlation was found between the k-casein genotype and reproductive characteristics at birth, favoring AB females. This correlation resulted in an increase in both litter weight and litter size. The genotype of the females were found to have no impact on weight gain, the survival of the young between birth and weaning, or the litter weight at weaning after the standardization of the litter size at birth (Bolet et al., 2007).

In cows, although κ-casein genotypes were not associated with reproductive performance (Tsiaras et al., 2005; Hamza et al., 2010), they were associated with thermal resistance, shorter coagulation times, better curdles, and micelles of diverse sizes (Schaar et al., 1985). It was also correlated with milk production and milk protein percentage (Mohammadi et al., 2013).

### Insulin receptor substrate-1 (IRS1)

The *IRS1* gene (ENSOCUG00000007187) in rabbits is found on chromosome 7 (168,540,584 − 168,544,330 R), and it has two exons and one transcript. The *IRS1* gene belongs to the IRS family, which is known to control postnatal growth (Pete et al., 1999; Baroni et al., 2001), and knocked-out IRS1 mice exhibit severely retarded growth (Araki et al., 1994). The *IRS1* gene was reported to affect growth and carcass traits in rabbits (Helal et al., 2022; Megahed et al., 2023; Safaa et al., 2023), sheep (Zhou et al., 2021), pigs (Niu et al., 2009), and beef cattle (Wu et al. 2014b).

In rabbits, only one study investigated the association between the *IRS1* gene polymorphism and reproductive traits. The c.189G > T SNP was genotyped in NMER rabbits using RFLP-PCR (Megahed et al., 2023), where the G allele was predominant with a frequency of 0.81. The GT genotype was significantly superior (*p* < 0.05) to the other genotypes in reproductive traits, including litter size at birth, live size at birth, litter weight at birth, and total litter weaning weight.

## Other related genes

In addition to the aforementioned genes, which may serve as markers related to the prolificacy of rabbits, many other genes have been reported to influence the reproductive traits of other species, but they have not been studied in rabbits yet.

### Follicle-stimulating hormone receptor gene (FSHR)

The *FSHR* gene (ENSOCUG00000011234) in rabbits resides on chromosome 2, (137,267,219 − 137,433,027 F). It has ten exons and four introns. The product of this gene is FSH hormone that controls reproductive traits, FSH is mediated by FSHR, which belongs to G-protein-coupled receptors family (Livshyts et al., 2009). FSHR activation by FSH promotes the growth and development of ovarian follicles. FSH stimulates the secretion of estrogen by the granulosa cells within the follicles, leading to the maturation of the oocytes within the follicles. It also plays a central role in spermatogenesis (Zhang et al., 2012). Moreover, *FSHR* also controls genes associated with lipogenesis (Yang et al., 2018).

SNPs in the *FSHR* gene were associated with reproductive diseases in humans (Gu et al., 2010). In Chinese sheep, SNPs detected in the 5′ regulatory region of the *FSHR* gene were associated with litter size (Chu et al., 2012; Guo et al., 2013). Similar findings were reported in other breeds (Pan et al., 2014; Abdel-Rahman et al., 2019). Also, *FSHR* polymorphisms were found to have a significant effect on the total born number and the number of born alive in Erhualian and Yorkshire sows (Shujun et al., 2002). In goats, significant effects of *FSHR* gene polymorphisms on litter size (Zhu et al., 2007). In poultry, the *FSHR* polymorphisms were linked to egg production in chickens (Li et al., 2019), and ducks (Li et al., 2019).

### Estrogen receptor 1 gene (ESR1)

The ESR1 gene (ENSOCUG00000004829) in rabbits resides on chromosome 12 (141,843,408 − 142,163,884 F) and has 13 exons and two transcripts. The encoded protein is a transcription factor that is activated by estrogen. The *ESR1* is necessary for the sexual differentiation of females and fertility. In the human testis, *ESR1* expression was associated with spermatogonia, spermatocytes, and round spermatids (Fietz et al., 2014). In rabbits, semen abnormalities and defects in spermatogenesis increased due to the absence of testis *ESR1* expression (Dewaele et al., 2022). Moreover, there is an SNP detected in *the ESR1* intron region in human females (T/C) is involved in sterility and preeclampsia (Molvarec et al., 2007).

To our knowledge, no studies addressed the association between *ESR1* gene polymorphisms and reproductive traits in rabbits. In Large White and Landrace Herd pigs, polymorphisms in the *ESR1* gene were correlated with litter size (Muñoz et al., 2007). However, in the Chinese-European pig line, polymorphisms of the *ESR1* gene were not associated with litter size but were associated with the total number of piglets born (Muñoz et al., 2007).

### Estrogen receptor 2 gene (ESR2)

The *ESR2* gene (ENSOCUG00000005083) in rabbits is found on chromosome 20 (11,921,224 − 11,973,480 R) and has 8 exons and two transcripts. Although both *ESR1* and *ESR2* are encoded for estrogen receptors and mediate estrogen signaling pathways, they exhibit differences in tissue distribution, ligand specificity, physiological functions, and gene regulation. *ESR2* is essential for regular ovulation efficiency and the development of peri-implantation embryos (Kowalski et al., 2002; Noguera et al., 2003).

In Chinese-European sows, an SNP located on the *ESR2* gene was tested for association with either the total number of piglets born or the NBA, but no significant association was detected (Muñoz et al., 2007).

### Insulin-like growth factor I (IGF1)

The *IGF1* gene (ENSOCUG00000014689) in rabbits resides on chromosome 4 (81,676,119 − 81, 750,443 R), it has 7 exons and 4 transcripts. Functionally, the IGF1 gene is linked to insulin, but it exhibits a significantly stronger growth-promoting action. On the reproductive level, it plays a role in sex-specific development or reproductive performance.

The female mice with mutant *IGF1* were found to be infertile (Neirijnck et al., 2019). Also, *IGF1* boosts the stimulatory action on progesterone synthesis, estradiol/aromatase, luteinizing hormone receptor, and inhibin-alpha (Adashi et al., 1985). In Sarda sheep, an SNP located in the *IGF1* gene was associated with an increased fertility rate (Sebastiano et al., 2020). Also in bovine granulose cells, FSH works in tandem with IGF1 to increase cell number and aromatase expression (Silva et al., 2009). In Iranian Holstein cows, the gene was sequenced, but SNP did not show any effect on milk production or reproduction traits (Abdolmohammadi and Zamani, 2014). Also, two SNPs within the *IGF1* gene were related to body condition scores at calving (Mullen et al., 2011).

### Growth hormone gene (GH)

The *GH* gene (ENSOCUG00000004341) in rabbits resides on chromosome 19 (48,725,660 − 48,727,208 R), it has six exons and three transcripts. GH is usually associated with growth, but it has different roles in mediating nutritionally-induced changes in follicular development (Downing and Scaramuzzi, 1991). Moreover, it has different effects on ovaries as it induces the development of small follicles in the gonadotrophin-dependent stages and stimulates oocyte maturation (Silva et al., 2009).

Polymorphisms in the *GH* gene were also correlated with semen characteristics in rabbits (Khalil et al., 2021). Associations between GH gene polymorphisms and prolificacy traits in rabbits have not been studied so far.

The association between GH gene polymorphism and litter size was reported in goats (Lan et al., 2007), pigs (Franco et al., 2005), and bovines (Joudrey et al., 2003). The effects of polymorphisms in the *GH* gene on prolificacy traits were studied in Matou and Boer goat breeds. Two SNPs were detected and linked to the reproductive characteristics and superovulation response (Zhang et al. 2011b).

### Inhibin subunit alpha gene (INHA)

The *INHA* gene (ENSOCUG00000011353) in rabbits resides on chromosome 7 (160,759,947 − 160,763,674 F), it has three exons and one transcript. This gene is one of the five key genes in the transforming growth factor-β (*TGFβ*) superfamily and plays an important role in reproductive functions (Welt et al., 2002). Alpha subunits in inhibin A and B protein complexes occur through proteolytic processing of the encoded preproprotein. These complexes inhibit the pituitary gland secretion of follicle-stimulating hormone (FSH). Inhibins play significant roles in corpus luteum formation and ablation (Han et al., 2023). Inhibins have also been associated with regulating different physiological functions, such as hormone secretion, immune response, cellular growth, and apoptosis (Nikitkina et al., 2021; Bao et al., 2023).

Polymorphisms of the *INHA* gene have not been studied in rabbits, although they have been extensively studied in other species. Changes in the *INHA* gene were associated with changes in sperm parameters in humans (Rafaqat et al., 2020). In chickens, SNPs found in the *INHA* gene were associated with ovulation rate and egg number (Cui et al., 2019). In Chinese Holstein cows, superovulation traits were found to be associated with an INHA gene polymorphism (Tang et al., 2011). The relationships between *INHA* polymorphisms and reproductive traits have been extensively studied in pigs and goats (Lin et al., 2006; Haroon et al., 2019; Chen et al., 2022). In Suhuia pigs, polymorphism at the 5’ untranslated region on the *INHA* gene was associated with litter size (Liu et al., 2017). Furthermore, follicular cysts were linked to an insertion/deletion mutation in Large White sows. (Li et al., 2016). In goats, the polymorphisms of *INHA* were studied in three different breeds (Xinong Saanen, Guanzhong, and Boer), and one SNP was significantly associated with litter size in all of them (Hou et al., 2012). Many studies confirmed the association between INHA polymorphisms and prolificacy and litter size traits in goats (Harris et al. 2005; Chu et al. 2007b; Wu et al. 2009; Pillai and Venkatachalapathy 2020).

### Leptin gene (LEP)

The *LEP* gene (ENSOCUG00000010189) in rabbits is placed on chromosome 7 (16,079,679 − 16,081,684 R), it has three exons and one transcript. Leptin also controls food intake and energy balance. As leptin controls feed intake, most of the work were directed to the association with growth traits. In Tianfu black rabbits the expression of leptin mRNA was associated with meat quality. Also, Leptin polymorphisms were associated with carcass and meat quality traits in beef cattle (Schenkel et al., 2005) and in Simmental-cross steers (Tian et al., 2013). In rabbits, the polymorphisms in the *LEP* gene were associated with growth (Helal et al., 2022), carcass (Radwan et al., 2023), and meat quality traits (Migdal et al., 2018). Nevertheless, no studies addressed the association between *LEP* gene polymorphisms and prolificacy traits in rabbits. On the other hand, the *LEP* gene polymorphism was associated with litter size in pigs (Chen et al., 2004; Terman, 2005; Wang et al., 2011), goats (Wang et al., 2011; Alim et al., 2019), sheep (Mahmoud et al., 2014; Younis et al., 2019), as well as the association with reproductive performance in cows (Chebel and Santos, 2011; Trakovická et al., 2013).

## Implications and future prospective

The current work demonstrated a summary of genes that have been associated with reproductive traits in rabbits including *PGR, FSHβ, OVGP1, Timp1, PRLP, CSN3, and IRS1 genes.* Some others are associated with the same traits in other animals, including *ESR1, ESR2, IGF1, GH. INHA, and LEP genes*. However, several other crucial genes were not encompassed in the present study. It is necessary to conduct further research on these genes. These genes include *GOT1A/Kiss1, LIF, LHX4, GNRHR, MAN2B2, MUC4, ROPN1, RBP4, AKR1C2, EPOR, LCK, and MSTN* genes. These genes also seem important for prolificacy traits in rabbits, as they were associated with similar traits in one or more other species.

## Conclusion

Finally, in this review, we summarized genes that were studied in rabbits and some genes that need to be studied in the near future. Here it should be emphasized that all those genes are candidate genes, and breed-specific effects may be altered by the cited results of other breeds. Furthermore, the interaction of the genetic architecture of reproductive traits highly complex. The current work indicates that big efforts are still needed. Additional candidate genes for prolificacy traits in rabbits should be investigated in different breeds under different conditions, this will allow for a much better understanding of the complex pathways underlying prolificacy and reproductive traits in rabbits.

## Data Availability

Not applicable.
